# 

**Published:** 1989-11

**Authors:** 


W1 BR323
V.104 NO.4 1989
C.01--------SEQ: B33740000
TI: BRISTOL MEDICO-CHIRURGICAL
JOURNAL

### BRISTOL MEDICO-CHIRURGICAL JOURNAL

VOLUME 104(iv) NOVEMBER 1989

Impressions of a Bristol President
Carey Coombs
Hospitals and the White Paper
Inguinal hernia waiting lists
Self inflicted injury
From our correspondents
Orthopaedic Reports etc.

NATIONAL LIBRARY
JAN 29 1990
OF MEDICINE

THE MEDICAL JOURNAL OF THE SOUTH WEST

